# Seroprevalence of viral hepatitis in jaundiced pregnant women in a teritiary care hospital

**DOI:** 10.1186/1471-2334-14-S3-E32

**Published:** 2014-05-27

**Authors:** Rudrapathy Parthiban, Raveendran Vinod, Saraswathi, Javangula Umarani

**Affiliations:** 1 Sri Venkateshwaraa Medical College Hospital & Research centre, Puducherry, India

## Background

Viral hepatitis represents an important health problem in the developing countries. The objective of this study is to determine the seroprevalence of viral hepatitis pattern in jaundiced pregnant women.

## Methods

A total of 128 jaundiced pregnant women admitted as in-patients were included in the study. The test samples were screened for viral hepatitis markers; HBsAg, Anti-HAV (IgM), Anti-HCV, Anti-HDV, Anti-HEV (IgM) by commercial ELISA kits. For HBsAg positive women, Anti-HBc IgM, HBeAg and Anti-HBe were done. HBV DNA analysis was done by polymerase chain reaction.

## Results

Of the 128 test serum samples screened, 53.1% were positive for HBsAg, 15.62% were positive for HEV IgM and 3.25% were positive for HAV IgM. None of them were positive for Anti-HCV & Anti-HDV. Among the 68 HBsAg positive pregnant women, the prevalence of HBeAg, anti-HBe, and anti-HBc were 54%, 45.6% and 89%, respectively. Of the 31, HBeAg negative women, 11 had only anti-HBe, 9 were positive for anti-HBe and HBV DNA and 11 were negative for anti-HBe and HBV DNA.

## Conclusion

The present study conducted has evolved a comprehensive picture on the virological pattern of viral hepatitis in jaundiced pregnant women. Hepatitis B virus (53.1%) seems to be the major causative agent followed by Hepatitis E virus (15.62%) in the 128 study subjects. Authenticated documentation on pregnancy hepatitis, maternal and fetal outcome in symptomatic viral infected mothers are essential features to plan and implement health measures package in a country.

